# A case of atypical type A thymoma variant

**DOI:** 10.1186/s40792-016-0245-3

**Published:** 2016-10-21

**Authors:** Masaki Hashimoto, Shigeki Shimizu, Teruhisa Takuwa, Yoshitane Tsukamoto, Tohru Tsujimura, Seiki Hasegawa

**Affiliations:** 1 Department of Thoracic Surgery, division of Surgery, Hyogo College of Medicine, 1-1 Mukogawa-cho, Nishinomiya, Hyogo 663-8501 Japan; 2 Department of Pathology, Kinki University of Medicine, Osakasayama, Japan; 3 Department of Pathology, Hyogo College of Medicine, Nishinomiya, Japan

**Keywords:** Thymoma, Atypia, Median sternotomy

## Abstract

**Background:**

An atypical type A thymoma variant was newly added to the WHO classification of type A thymoma family in 2015.

**Case presentation:**

A 72-year-old female was present a large round mass in the anterior mediastinum. The radiological examination led to a preoperative diagnosis of non-invasive thymoma. Tumor resection was undertaken via median sternotomy. Complete removal of the mediastinal tumor was achieved. Pathological examination revealed that the tumor cells were spindle- and oval-shaped with atypia. Immunohistochemical work-up revealed that the tumor was type A thymoma. On the basis of these findings, the tumor was finally diagnosed to be an atypical type A thymoma variant.

**Conclusions:**

Preoperative diagnosis as atypical type A thymoma variant based on radiological examination is difficult. In case of atypical type A thymoma variant, a careful postoperative systemic follow-up should be done.

## Background

In general, type A thymoma is recognized as a benign tumor with excellent prognosis [1]. However, several authors have reported type A thymoma showing atypical features with postoperative tumor relapse [2–4]. On the basis of these reports, atypical type A thymoma variant was added to the type A thymoma family as a small subset of aggressive tumors [5, 6]. Here, we present the case of an atypical type A thymoma variant.

## Case presentation

**Fig. 1:**
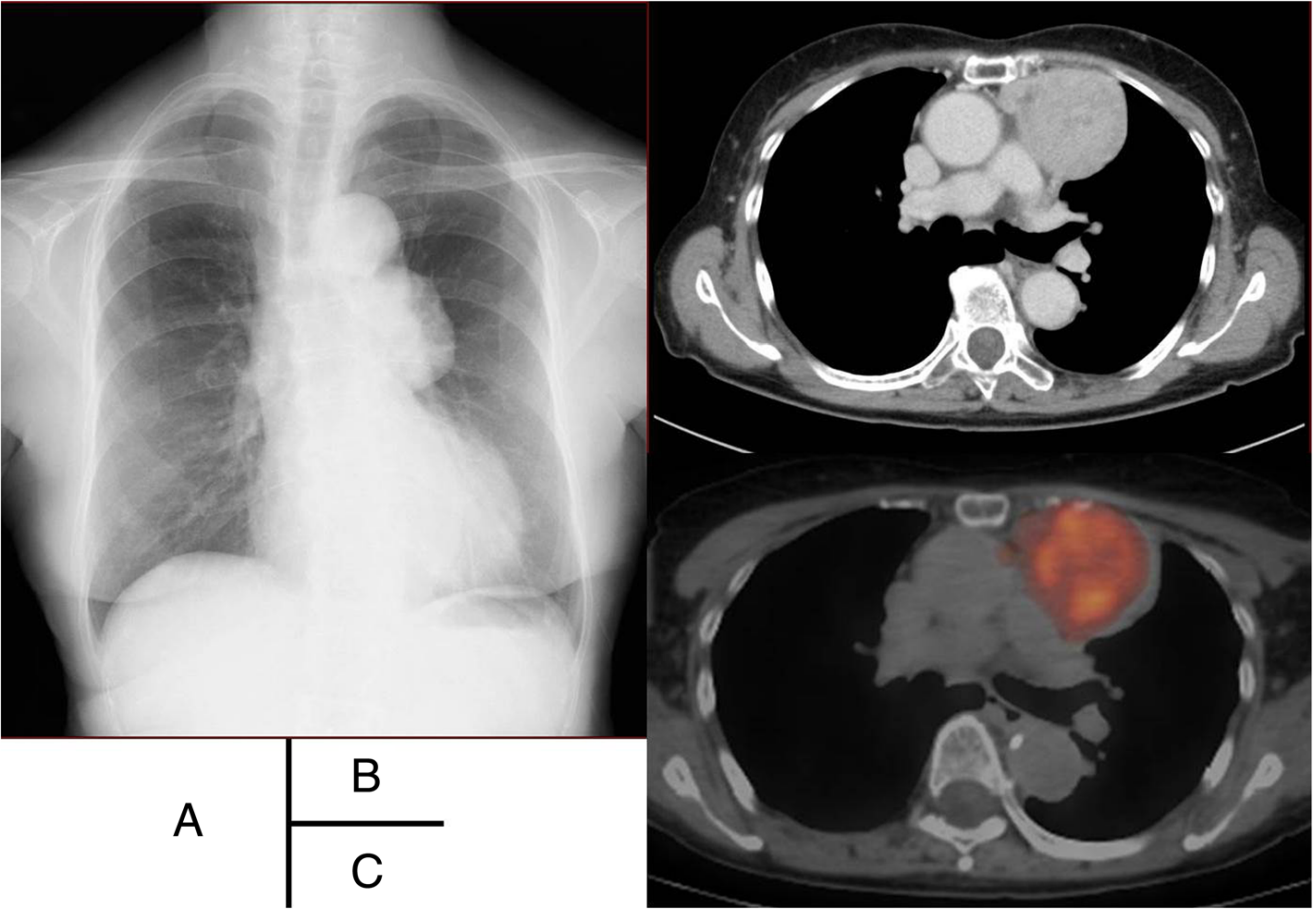
Chest X-ray showing an abnormal shadow in the mediastinum (**a**). Contrast-enhanced chest computed tomography showing a large round mass not invading the surrounding organs in the anterior mediastinum (**b**). Fluorodeoxyglucose positron emission tomography showing a mild hypermetabolic mass in the anterior mediastinum (**c**)

A 72-year-old female presented with a mediastinal mass that was incidentally detected on chest X-ray (Fig. 1a). Chest computed tomography (CT) revealed a large round mass of 7.7 cm in diameter in the anterior mediastinum. Contrast-enhanced CT (CE-CT) revealed that the mediastinal mass was not invading the surrounding organs (Fig. 1b). Fluorodeoxyglucose positron emission tomography (FDG-PET) showed mild uptake, with a maximum standardized uptake value (SUVmax) of 3.5 at the mediastinal mass (Fig. 1c). Abnormal uptake in other organs, which may lead to a suspicion of distant metastasis, was not detected. She did not present with myasthenia gravis or any other autoimmune diseases.

Our preoperative diagnosis was a non-invasive thymoma, and she underwent tumor resection via median sternotomy. The operative findings revealed that the tumor did not invade the surrounding organs, and we could easily dissect the tumor.

**Fig. 2:**
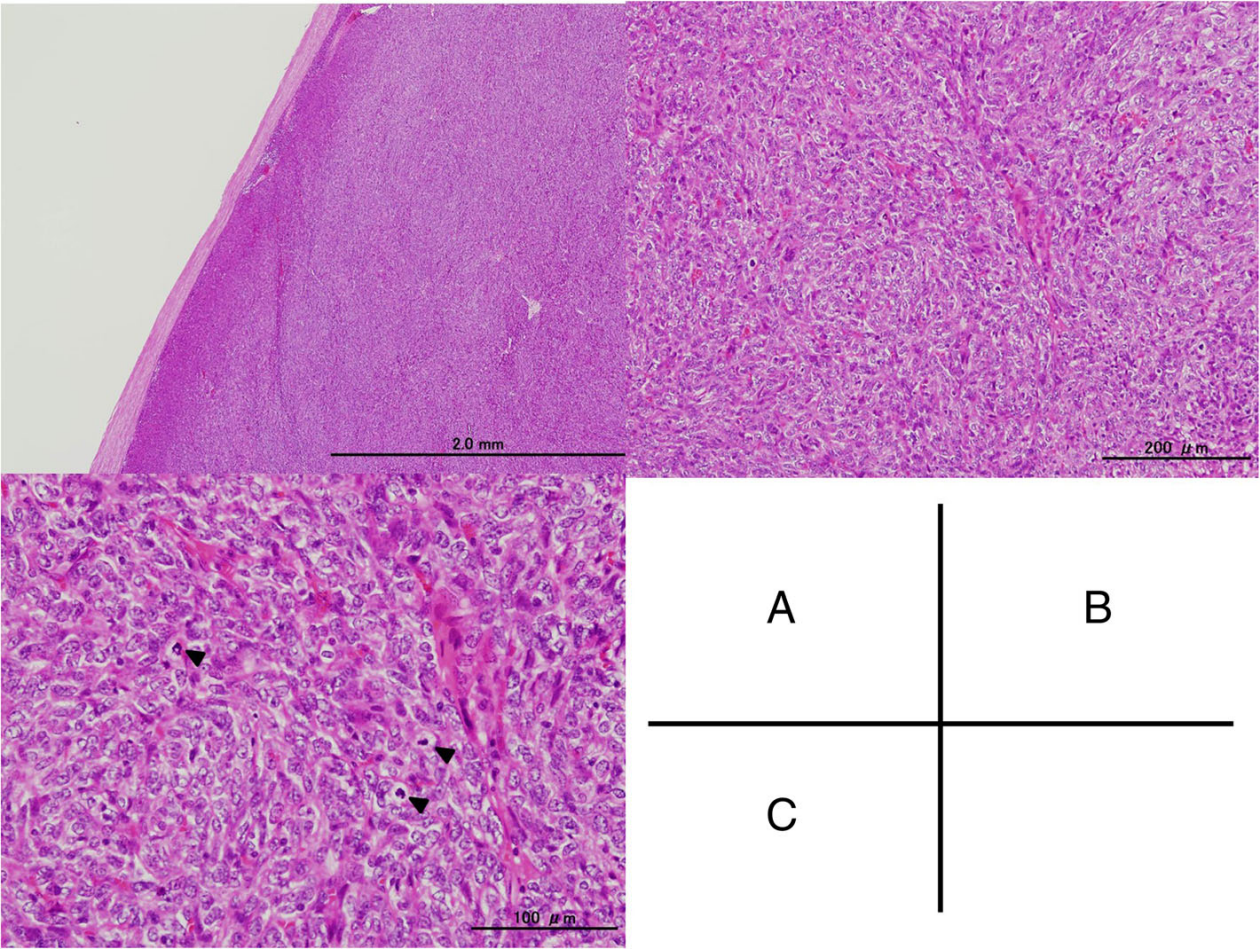
Microscopic findings on hematoxylin–eosin staining showing encapsulated (**a**), the spindle- and oval-shaped tumor cells with hypercellurality, moderate atypia, and high mitotic activity (*arrowhead*) (**b, c**)

**Fig. 3:**
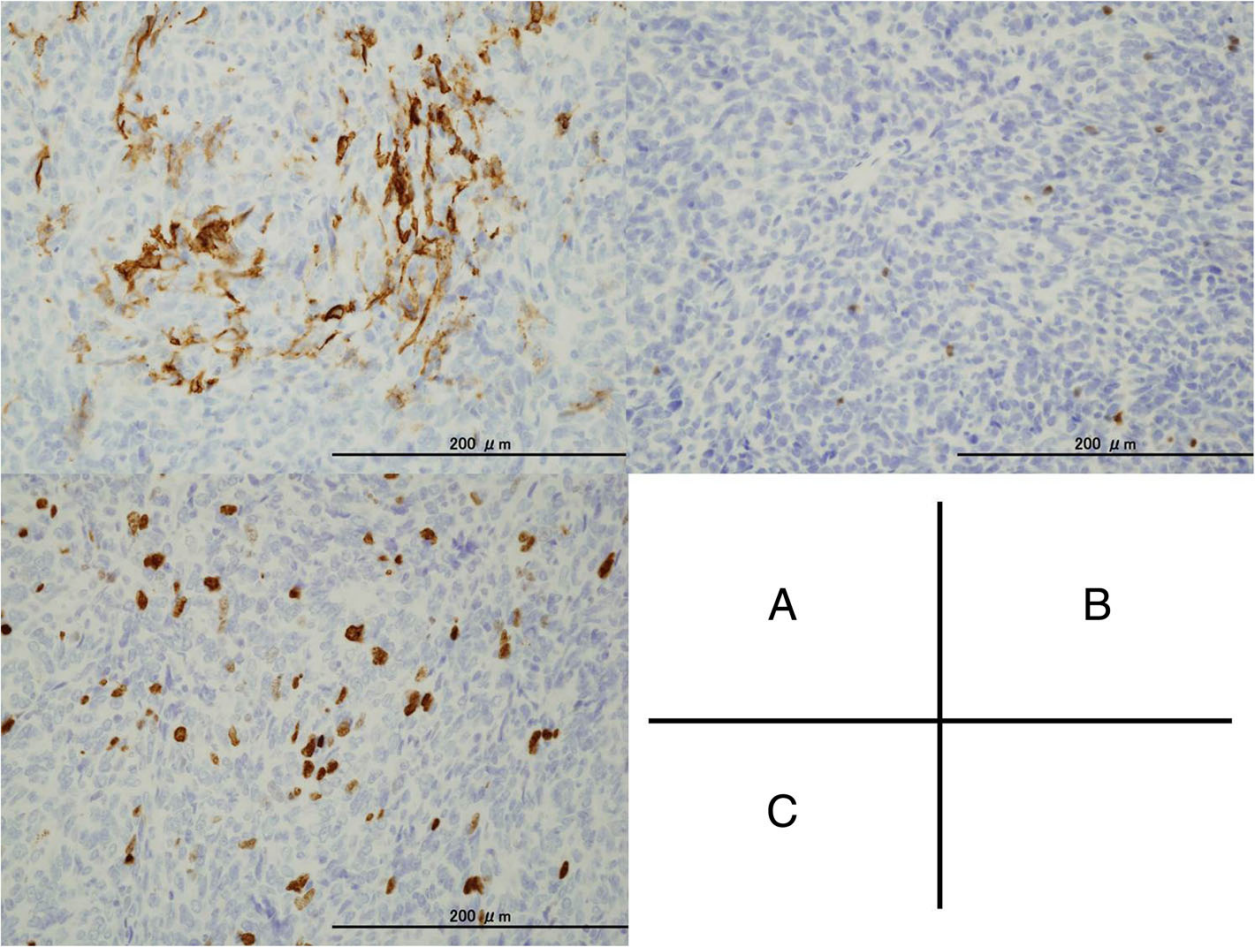
Microscopic findings on immunohistochemical staining aberrant expression for CD20 (**a**). Positive for terminal deoxynucleotidyl transferase in a few tumor cells (**b**). Ki-67 labeling index was 23.3 % (**c**)

Pathological findings showed that the tumor was surrounded by a fibrous capsule (Fig. 2a), and its cells were spindle- and oval-shaped (Fig. 2b). A hemangiopericytoma-like vascular pattern was present. The tumor cells showed mild atypia, hypercellularity, and moderate mitotic activity (8–10 mitoses per 2 mm^2^). Necrosis was absent (Fig. 2c). A significant reticulin network was found around individual tumor cells. In the immunohistochemical work-up, the tumor cell was positive for AE1/AE3, p40, and CK5/6 and negative for CD5 and c-kit. CD20 expression was detected in some tumor cells (Fig. 3a). In the terminal deoxynucleotidyl transferase (TdT) staining, a few TdT + T cells were found in the tumor (Fig. 3b). The Ki-67 labeling index was 23.3 % (Fig. 3c). On the basis of these findings, the tumor was finally diagnosed to be an atypical type A thymoma variant.

Her postoperative course was uneventful, and she was discharged on foot 12 days after surgery. She is doing well without recurrence until last follow-up at 15 months after operation. We continue to conduct careful postoperative follow-up.

### Discussion

An atypical type A thymoma variant was added to the WHO classification of type A thymoma family in 2015 [5] based on several reports about type A thymoma with oncological aggressive behavior and tumor relapses [2–4]. Pathological findings present the most specific feature of atypical type A thymoma. These are as follows: (1) mild to moderate nuclear atypia, (2) increase in mitotic activity, and (3) a scattered foci of necrosis. These findings are usually present in type B3 thymoma rather than the conventional type A thymoma. Vladislav and coworkers described that the frequency of type A thymoma with these aggressive behavior was 3.8 % (23/600 cases) [2]. In this case, the patient presented with mild atypia, hypercellularity, and high mitotic activity, which was 8–10 mitoses per 2 mm^2^. This distribution was denser than conventional type A thymoma where the count is usually <4 mitoses per 2 mm^2^.

On the other hand, specific finding of radiological examination was still unknown. CECT could show the possibility of tumor invasiveness to surrounding organ, but it would not reflect of morphological behavior. Recently, PET-CT has been known as a useful examination about thymic epithelial tumors, especially thymoma [7, 8]. Park and coworkers showed that significant relationship was observed between SUVmax and WHO classification, mean SUVmax in low-risk thymoma (A, AB, and B1) was 3.43, and high-risk thymoma (B2 and B3) was 4.42 [7]. However, in this case, SUVmax was 3.5; it might not be able to predict the morphological behavior. As these results, neither CECT nor PET-CT contributes preoperative definitive diagnosis of atypical type A thymoma variant.

Vladislav and coworkers showed that tumor relapse, including distant metastases, occurred in 43 % (10/23 patients) cases of type A thymoma showing atypical features [2]. This relapse rate is much higher than that of conventional type A thymoma.

The mean duration of time to metastases was 39.7 months (7–107 months). Of these 10 patients, lung metastasis was found in 5, and liver metastasis in 4. Regarding surgical margin and postoperative tumor metastasis, contrary to logic, development of metastatic disease was observed more frequently in the patients with negative surgical margin than positive or close (<1 mm) surgical margin. And they also described that only the presence of necrosis is a predictive factor of tumor relapse, including distant metastases, and no other factors such as the stage of diagnosis, tumor size, nuclear shape, nuclear variability, and mitotic activity correlate with tumor relapse. These suggested that postoperative follow-up about not only intrathoracic cavity but also extrathoracic cavity is essential.

Ki-67 is a well-known histological marker of proliferation used as an index of biological aggressiveness in various solid tumors. It has already been reported that Ki-67 labeling index (LI) correlates with the tumor aggressiveness in thymic epithelial neoplasm [9]. In that report, Ki-67 LI in type A thymoma was 0.3–11.0 % (median 3.0) and in thymic carcinoma was 12.2–43.3 % (median 23.2 %) [9]. Ki-67 LI in the present case was 23.3 %, which suggests that the aggressiveness of this tumor is similar to that of thymic carcinoma. The correlation between Ki-67 LI and tumor relapse in thymoma is not confirmed, but careful postoperative follow-up may be essential in atypical type A thymoma variant.

## Conclusions

Here, we presented the case of an atypical type A thymoma variant. It is notable that radiological examination did not contribute to the definitive diagnosis of the atypical type A thymoma variant. When we encounter the case of atypical type A thymoma variant, careful postoperative systemic follow-up should be conducted.

## Abbreviations

CT: Computed tomography
TdT: Terminal deoxynucleotidyl transferase
